# Histomorphometric evaluation of bone healing in rabbit fibular osteotomy model without fixation

**DOI:** 10.1186/1749-799X-3-4

**Published:** 2008-01-29

**Authors:** Marcos A Matos, Francisco P Araújo, Fabio B Paixão

**Affiliations:** 1 Department of Surgery, Division of Orthopaedics, Bahia School of Medicine and Public Health, 275 D. João VI Ave., Salvador-Bahia, Brazil

## Abstract

**Background:**

Animal models of fracture consolidation are fundamental for the understanding of the biological process of bone repair in humans, but histological studies are rare and provide only qualitative results. The objective of this article is to present the histomorphometric study of the bone healing process using an experimental model of osteotomy in rabbit fibula without interference of synthesis material.

**Methods:**

Fifteen rabbits were submitted to fibular osteotomy without any fixation device. Groups of five animals were submitted to pharmacological euthanasia during a period of one (group A), two (group B) and four weeks (group C) after osteotomy. Histomorphometric evaluation was performed in the histological sections.

**Results:**

During week one there was intense cellularity (67/field), a large amount of woven bone (75.7%) and a small amount of lamellar bone (7.65%). At two weeks there was a decrease in woven bone (41.59%) and an increase in lamellar bone (15.16%). At four weeks there was a decrease of cellularity (19.17/field) and lamellar bone (55.56%) exceeded the quantity of woven bone (31.68%).

**Conclusion:**

Histomorphometric (quantitative) evaluation of the present study was shown to be compatible with bone healing achieved in qualitative experimental models that have been commended in the literature.

## Background

The use of animal models to study fracture consolidation is particularly useful to answer questions related to the most effective way to treat human beings. Most of the information concerning the molecular and cell biology of bone repair in man had its originated from experimental models[1,2].

Several models described in the literature are useful for the study of bone consolidation, but histological studies are rare and provide only qualitative results [1-4]. The majority of them, however, use synthesis material to fix the fracture focus, and this type of device interferes in the natural biological process[5]. Moreover, the histomorphometric (quantitative) pattern of natural fracture repair is not yet sufficiently studied in the literature.

The purpose of this article is to present a histomorphometric study of the biological bone repair process using an experimental model of osteotomy in the rabbit fibula without interference of fixation devicies.

## Methods

This study conformed to the Guiding Principles on the Care and Use of Laboratory Animals, and was approved by the Research Ethics Committee at Bahia School of Medicine and Public Health.

Fifteen skeletally immature (epiphyseal ring still open and aged 1.5 months) albino New Zealand male rabbits (*Oryctologus cuniculus*) with an average weight of 975 g (± 103.31) were used. Animals were divided into three groups, assigned A, B and C (five animals in each group). They were kept in a bioterium and were housed in individual cages during the entire study period with water and chow diet *ad libitum*.

All the the animals were submitted to the rabbit osteotomy model reported in 2001[5]. Food was suspended eight to ten hours before anesthesia was administered. To decrease the vagal tonus, each animal received 0.2 mg/kg dose of atropine sulphate by intramuscular injection. Animals were anesthetized by intraperitoneal injection of ketamine (25.0 – 30.0 mg/kg of body weight) and intramuscular injection of diazepam (5.0 to 10.0 mg/kg of body weight).

By the aseptic condition technique, the fibula of each animal was accessed by a lateral approach of approximately 5 mm in the right pelvic limb. After skin and subcutaneous tissue division, the fibular muscle fascia and periosteum were opened and dissected from the cranial portion of the fibula. Shaft osteotomy was performed on the cranial portion of the exposed fibula, using an electric saw with a standardized blade (10.0 mm wide and 0.5 mm thick). The incision was closed in layers, using absorbable 5-0 polyvicryl sutures for the fascia and 5-0 mononylon sutures for the skin (no patch was used).

After one (group A), two (group B) and four (group C) weeks, animals were anesthetized and killed with a 2 ml intracardiac injection of potassium chloride. The fibula of each animal was removed, dissected from the surrounding soft tissue, and fixed in 10% formalin for microscopic evaluation. Formalin-fixed bones were decalcified with 7.5% nitric acid, embedded in paraffin, and longitudinally sectioned. Histological sections (7 μm thick) were stained with hematoxylin and eosin prior to optical microscope examination.

Three histological sections were analyzed for each animal. After the cuts had been chosen, a preliminary analysis was performed at 100× magnification in order to determine the area of the callus, defined by the regions associated with significant periosteal thickening, i.e. the area where the cortical bone thickness had more than doubled. Histomorphometric evaluations of all microscopic fields were performed using a test eyepiece reticule with 10 parallel lines and 100 points containing a grid with a total area of 10.500 μm^2 ^(Zeiss 23-9901) at a magnification of 200×. The associated histomorphometric parameters are taken from a previous report by Parfitt et al.[6] and Compston[7]. The parameters were presented in percentage of total callus and not in absolute numbers (area) to homogenize results of different bone callus areas (table 1).

**Table 1:** Description of the histomorphometric parameters.

Histomorphometric parameters	Description
Tissue volume	Total callus area inclusive of all tissue both within and outside the original bone cortices.
Woven bone (area, %)	The fraction of the tissue volume which is occupied by woven bone (primary or immature tissue). Figures 2 and 3.
Lamellar bone (area, %)	The fraction of the tissue volume which is occupied by lamellar bone (secondary or mature tissue). Figures 1 and 3.
Periosteal fibrosis (area, %)	The fraction of the tissue volume which is occupied by fibrous tissue. Figure 2.
Marrow fibrosis (area, %)	The fraction of the tissue volume which is occupied by fibrous tissue on the marrow area. Figure 1.
Cellularity (number, mean/field)	The number of cells.

Tissues that represented callus maturation, woven bone (immature) and lamellar bone (mature) were quantified; as well as those that represented soft tissues intimately related to the bone reparative process (marrow and periosteal fibrosis). Tissues that represented the inflammatory or unspecific reparative process, such as medullary fat, vessels, bone canals width, were not quantified, and represented a minimum percentage in the callus area[6,7]. No techniques were used for identifying the cellular population (type), and only the total number of cells per field (within the the fibrosis, cartilage and bony tissues) was considered, but quantification of the tissues provides an indirect impression of which cellular elements would be acting in each phase.

Results are reported as mean ± standard deviation. Differences between groups were assessed using Kruskal-Wallis test (and Duns post-test when necessary) for independent non-parametric data and the level of significance was p < 0.05.

## Results and discussion

All animals survived to the end of the study. No wound infection or dehiscence was observed in the animals.

**Figure 1:**
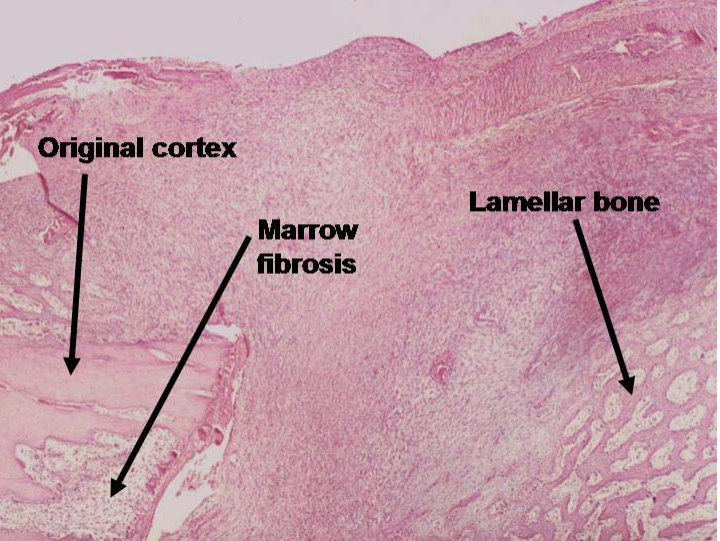
Microscopic appearance of the callus (longitudinal section) in the second week post fibular osteotomy in young rabbits, which shows marrow fibrosis, original cortex and a small amount of lamellar bone (HE).

**Figure 2:**
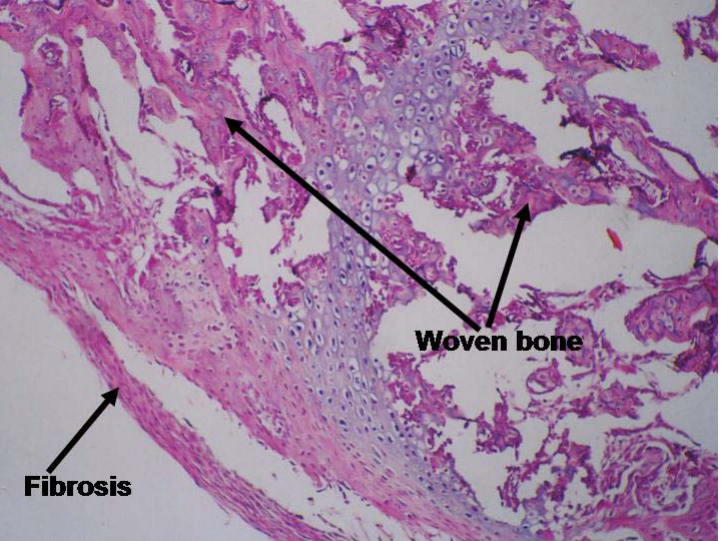
Microscopic appearance of the callus (longitudinal section) in the fourth week post fibular osteotomy in young rabbits, which shows periosteal fibrosis and woven bone (HE).

**Figure 3:**
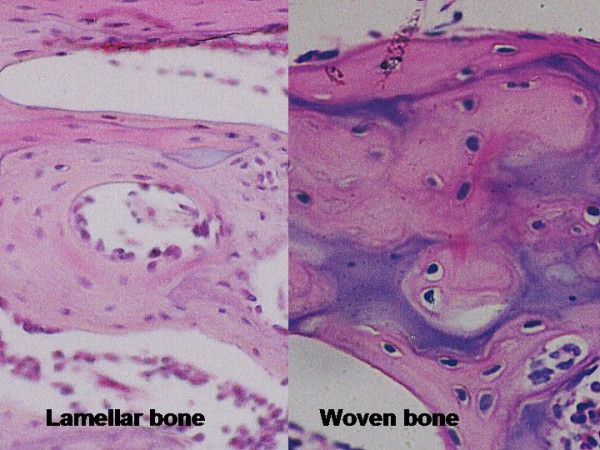
Histological cut showing details of lamellar bone concentrically organized and woven bone mixed with cartilage and calcified cartilage tissues (HE).

The results obtained are presented in Figures 1, 2 and 3, and Tables 2 and 3. An asterisk (*) was used to indicate statistically significant data compared to data of the first week.

**Table 2:** Woven bone and lamellar bone during fracture healing.

Reparative time	Woven bone (%)	Lamellar bone (%)
First week	75.70 (± 19)	7.65 (± 7)
Second week	41.59 (± 24) *	15.16 (± 13)
Fourth week	31.68 (± 21)*	55.56 (± 25)*

*p < 0.05

**Table 3:** Fibrosis volume, marrow fibrosis and cellularity during bone healing.

Reparative time	Periosteal fibrosis (%)	Marrow fibrosis (%)	Cellularity
First week	7.55 (± 6.2)	4.75 (± 4.4)	67.00 (± 20)
Second week	4.66 (± 4.4)	12.67 (± 10.4)	22.50 (± 12)*
Fourth week	3.75 (± 3.5)	21.68 (± 6.4)*	19.17 (± 8)*

*p < 0.05

The animal fracture consolidation models are fundamental for the understanding of the biological process of bone repair in man[1-4]. From these models it is possible to propose new techniques to treat and accelerate fracture consolidation. Many variables, however, make it difficult to extrapolate experimental data. Selection of the experimental animal and the type of repair achieved with the model represent the most important variables[2,3].

Small rodents have primitive bone structures and do not have haversian systems[2] and although little is known about the importance of this anatomical difference between rodents and humans, this makes bone repair in these animals different from that seen in human beings[2]. Whereas rabbits, as well as caprines and dogs, have haversian systems that are similar to that of man, which is an important advantage in terms of extrapolation of results obtained with such animals for human bone repair[2].

Rabbits are the most popular animal models in health science, and most researchers are acquainted with them[5]. The conditioning and cost of these animals allow the use of moderate sized groups when several experimental protocols are used[5]. Unlike rodents, the rabbit's size allows multiple collections from the same bone for testing biomechanical or histopathological properties testing[5].

Another essential question concerns the use of synthesis materials for fixation of the fracture produced in the model. The use of these devices interferes with the natural consolidation and does not allow periosteous (secondary) bone callus to be obtained, thus having a negative influence on analysis of results[5]. With rabbits it is possible to use external or internal fixation by means of plates and screws. In the model that was presented, however, it was possible to maintain the osteotomy that was performed in the fibula diaphysis without any fixation device, since the rabbit has a rudimentary fibula that is distally linked to the tibia, something that provided focus stability and made it possible to study bone repair in the most natural of all possible manners[5].

Histological studies of bone repair procedures are rare and usually provide qualitative results. The present study presents the histomorphometric quantitation of a three-stage bone repair using a model that allows the biological processes of periosteous repair to be observed, that is, the repair occurring without interference from the materials used in fracture fixation. This is a very important approach in the naturally occurring consolidation model presented.

The histological evaluation shows that the model can reproduce three different stages of bone repair. During Week 1 we observed an early repair stage characterized by multiple cells, a large amount of woven bone (bone in its primary structure) and a small amount of lamellar bone. According to Einhorn[1], the first consolidation phase, which extends until Day 10, is characterized by intense cell multiplication due to the action of inflammatory cytokines. At this point, proliferation of the fibrosis layer of the periosteum will occur with intense intramembranous formation of woven bone[2,4,7].

During the first hours following the fracture, the cell density will increase both in the external (periosteal) callus and the bone marrow[1,8,9]. This intense proliferation reaches its maximum around Day 3 and begins to decline before Week 2.

During the second stage, at two weeks (intermediate phase), the cell proliferation begins to decline and the primary bone associated with cartilaginous callus dominates the tissue. At this point, woven (new) bone starts being reabsorbed and replaced by lamellar (mature) bone, with emergence of hematopoietic, fatty and fibrous tissue in the marrow canal[2,4,7]. The amount of woven bone apparently reaches its maximum value near Week 2[2,4,7]. The present study model showed a callus compatible with this intermediate stage at Week 2, still showing high cellularity and a large amount of woven bone, and the beginning of an increase in the amount of lamellar bone.

Around Day 21 post-fracture (late stage), woven bone and cartilage could be seen in the periphery of the callus, although there was no more hypercellularity and the quantity of lamellar bone began to increase until Day 35[2,4,7]. The samples obtained during Week 4 in the present model evidenced a similar consolidation stage. At this point we found that the quantity of lamellar bone exceeded that of woven bone and intense marrow fibrosis with a marked decrease of cellularity could also be seen.

The overall histomorphometry of the callus, as presented in this study, is the primordial step for understanding bone repair, however, immunohistochemical and molecular biological studies are necessary for understanding the role of each tissue or cell type during this complex biological process.

## Conclusion

This study presents a new experimental model suitable for natural bone repair studies (without fixation devices) in which histomorphometric (quantitative) evaluation was shown to be compatible with the consolidation achieved in other experimental models that were commended in the literature.

## Competing interests

The author(s) declare that they have no competing interests.

## Authors' contributions

**MAM **conceived of the study, participated in the coordination and wrote the final version of the manuscript. **FPA **and **FBP **participated in the experimental procedures, data analysis and writing the manuscript. All authors read and approved the final manuscript.
